# Genetic diversity of *Gallus* sp. in Southeast Asia based on d-lopp: *In silico* study

**DOI:** 10.5455/javar.2023.j738

**Published:** 2023-12-31

**Authors:** Zultinur Muttaqin, Tike Sartika, Ferdy Saputra

**Affiliations:** Research Center for Animal Husbandry, Research Organization for Agriculture and Food, National Research and Innovation Agency of the Republic of Indonesia (BRIN), Cibinong Sciences Center, Cibinong, Indonesia

**Keywords:** D-loop, genetics, *Gallus* sp, *in silico*, Southeast Asia

## Abstract

**Objective:**

This study was conducted to analyze the matrilineal structure of *Gallus* sp*.* in Southeast Asia, especially in the D-loop region.

**Materials and Method:**

A total of 563 sequences from eight countries (Laos, Myanmar, Vietnam, Malaysia, Indonesia, Cambodia, the Philippines, and Thailand) in Southeast Asia are used in this study. Data collected from National Center for Biotechnology Information (NCBI) regarding the genus *Gallus* sp. in a Southeast Asian country. Data analysis was performed using MEGA 7.2 and DnaSP v6.

**Results:**

In the haplotype found in *Gallus* sp. in Southeast Asia, there are 89 haplotypes. Using a neighbor-joining (Nj) analysis, 89 haplotypes found three haplogroups for *Gallus* sp. in Southeast Asia. In Southeast Asia, the genetic diversity of the d-loop is exceptionally high, with a haplotype diversity value of 0.524 to 1.

**Conclusion:**

D-loop cannot be used as a specific marker for breeds or country-specifics.

## Introduction

Domestic chicken is widely used as a source of both meat and eggs all over the world. Asia has 53.3% of the world’s chicken population. From 1994 to 2021, FAO provides information that Indonesia ranks seventh as the meat-producing country under China and the US [1]. The population of chickens in Southeast Asia is 3,611,972,000 [2]. This indicates that the role of chickens is very important in people’s lives, especially in Southeast Asia.

In recent years, Indonesia has imported many breeds from other countries, especially from countries in Southeast Asia. Most of the chicken breeds in Indonesia come from Thailand, Vietnam, and Myanmar, with fighting advantages, e.g., Bangkok, Birma, Mangon, Saigon, Pakhoy, and Pama. Cockfighting is a hobby of Indonesian gamblers, and it is illegal in Indonesia. These fighting chickens are competed by some communities, and winning increases their price. Cockfighting in Bali is more than just gambling; it has an important role in culture [3]. However, at the community level, Indonesia cannot regulate cross-breeding between fighting chickens from other countries and Indonesian local chickens. Indonesia must adopt a closed breeding policy to maintain the purity of local chickens. Indonesia has implemented a closed breeding policy for Bali cattle and Madura cattle.

To maintain the genetic purity of local chickens, researchers need to conduct a study on the genetic diversity of chickens in Southeast Asia. D-loop is a mitochondrial DNA region that is the most commonly used for matrilineal studies. D-loop has high sequence variation and a moderate mutation rate [4]. Domestic chickens are the domestication of junglefowl in Southeast Asia [5]. Therefore, it is very important to analyze the matrilineal structure of *Gallus* sp. in Southeast Asia, especially in the D-loop region.

## Materials and Methods

The data used in this study were from the National Center for Biotechnology Information (NCBI). A total of 563 sequences from eight countries (Laos, Myanmar, Vietnam, Malaysia, Indonesia, Cambodia, the Philippines, and Thailand) in Southeast Asia are used in this study. All analyses in this study used a sequence length of 320 bp. The data were aligned by MUSCLE in MEGA 7.2 [6]. Haplotype diversity, number of haplotypes, and Fst distance were performed using DnaSP v6 [7]. Furthermore, using MEGA 7.2, the Fst distance was calculated using neighbor-joining (Nj) with 1,000 bootstrap replications.

## Results

**Figure 1.**
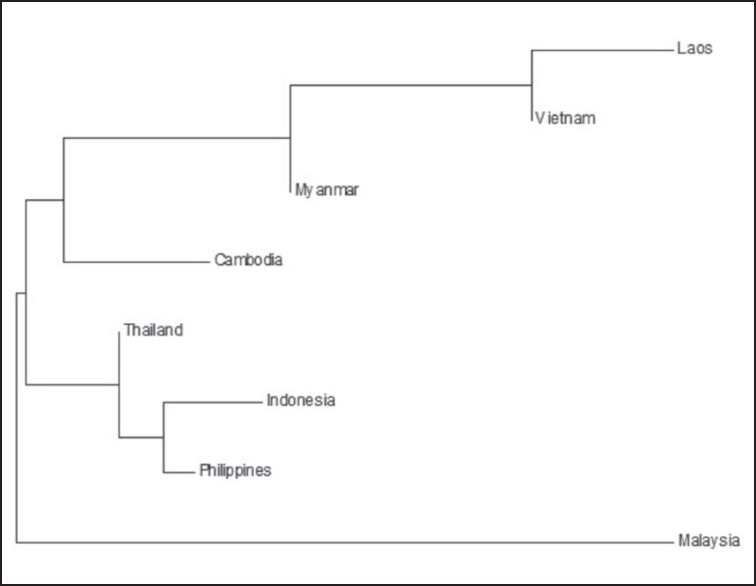
Neighbor-joining tree of *Gallus* sp. in Southeast Asia tree based on Fst genetic distance.

Analysis of data from NCBI resulted in 89 haplotypes. Indonesia had 44 haplotypes and high haplotype diversity (Hd = 0.902) in this study (Table 1). This happens because of the large amount of data and the various breeds of data used. The highest haplotype diversity (Hd = 1) was found in Thailand because three sequences of *Gallus* sp. in Thailand made three haplotypes. On the other hand, the least haplotype diversity (Hd = 0.524) was found in Malaysia. Nj-based Fst distance found a relationship *Gallus* sp in Laos, Vietnam, and Myanmar (Fig. 1). The Philippines and Indonesia had a strong relationship, and Thailand was in the same cluster as the Philippines and Indonesia. NJ showed Cambodia, Myanmar, Vietnam, and Laos in the same cluster. On the other hand, Malaysia was in an outgroup cluster.

**Table 1.** D-loop diversity of *Gallus* sp. in Southeast Asia.

Population	n	h	Hd
Laos	40	11	0.822
Myanmar	13	10	0.949
Vietnam	63	21	0.876
Malaysia	7	3	0.524
Indonesia	206	44	0.902
Cambodia	13	8	0.859
Philippines	22	14	0.948
Thailand	3	3	1

*n* = Number of sequence; *h* = number of haplotype; Hd = haplotype diversity

## Discussion

The sequence analyzed in this study contained information on chicken breeds in Indonesia, conducted by [8]. Bekisar is crossbred between the rooster of *Gallus varius* and the hen of *Gallus gallus domesticus* [9]. Based on the D-loop, Bekisar showed a similar haplotype to chickens from Laos and Myanmar. We found that pelung chicken in Indonesia had a specific haplotype. On the other hand, the Pelung haplotype has similar haplotypes to other Indonesian, Vietnamese, Myanmar, and Philippine chickens. This is possible because Southeast Asia is a center of chicken domestication. The haplotype diversity in Vietnamese chickens in Ha Giang Province ranged from 0.75 to 1, with a total haplotype of 25 [10]. This study suggested two centers of *Gallus* sp. in Southeast Asia, e.g., the north center (Laos and Myanmar) and the south center (Indonesia and Malaysia). Vietnam, the Philippines, Thailand, and Cambodia are the meeting centers of these two centers. The red junglefowl in the Philippines was closely related to Myanmar [11]. Herrera et al. [12] stated that South American chickens are more closely related to European chickens, while European chickens are more closely related to Southeast Asian chickens, which also have a high level of diversity. Wang et al. [13] supported the center of domestication occurring in Southeast Asia. Adaptation to high temperatures in domestic chickens is obtained from tropical red junglefowl [14]. The aggressiveness of roosters and the ability to exhibit cockfighting behavior in domestic chickens may be obtained from red junglefowl [15]. On the other hand, other studies have found that white feathers are more aggressive than red feathers [16]. Sartika et al. [17] stated that microsatellites cannot be used to distinguish eight native breeds of Indonesian chickens and argued that a microarray with many single nucleotide polymorphisms (SNPs) will be able to distinguish breed-specific. Zimmerman et al. [18] stated that significant SNPs are better than microsatellites at identifying groups in clustering methods and providing more accurate estimates of diversity. As a result, we propose additional research on finding and clustering genetic structures utilizing large SNPs.

## Conclusion

This research found 89 haplotypes with high haplotype diversity values in Southeast Asia. Furthermore, the D-loop cannot be used as a specific marker for breeds or country-specifics. A microarray analysis should be conducted to determine specific markers for breeds.

## List of abbreviations

NCBI, National Center for Biotechnology Information; NJ, neighbor-joining; SNPs, single nucleotide polymorphisms.
